# Wear behaviour of lithography ceramic manufactured dental zirconia

**DOI:** 10.1186/s12903-023-02974-4

**Published:** 2023-05-11

**Authors:** Ashwini Patil, Davidson Jebaseelan D, Daniel Bomze, Vasanth Gopal

**Affiliations:** 1 School of Mechanical Engineering (SMEC), VIT University, Vandalur-Kelambakkam road, VIT Chennai Campus, Chennai, Tamilnadu 600127 India; 2 Lithoz GmbH, Oberlaaer Straße 228, Vienna, 1100 Austria; 3 Lab for Texture and related studies, Department of Material Engineering, Indian Institute of Science, Bangalore, 560012 India

**Keywords:** Digital light processing (DLP), Lithography-based Ceramic Manufacturing (LCM), Tribology, 3-D surface roughness, Dental

## Abstract

**Objective:**

The study aims to evaluate the wear surface using 3D surface roughness and other material characterization of zirconia fabricated using photopolymerization based Lithography-based Ceramic Manufacturing (LCM).

**Method:**

LCM technology was used to fabricate zirconia specimens of size 10 × 10 × 2mm^3^. Scanning Electron Microscope, 3D–profilometer, X-ray Diffraction, and hardness test characterized the samples before and after wear and Coefficient of friction (COF) was monitored.

**Result:**

The COF was around 0.7 and did not differ much between the horizontally and vertically printed specimens. However, the surface roughness after wear for horizontally printed specimen was 0.567 ± 0.139 μm, while that for vertically printed specimen was 0.379 ± 0.080 μm. The reduced valley depth and the dale void volume were low for the vertically printed zirconia specimen, indicating lesser voids and low fluid retention. In addition, it was observed that the hardness value of the vertically printed sample was better. The scanning electron microscopic images and 3D surface profiles of the zirconia specimens depicted the surface topography and revealed the wear track.

**Conclusion:**

The study shows that zirconia fabricated using LCM technology possesses surface roughness of about 0.5 μm with no machining scars that are usually associated with CAD/CAM dentistry and also indicating agreement with clinically acceptable values for minimal surface roughness of dental restorations. Dental restorations using LCM fabricated zirconia redues the requirement of post-processing work flow that is part of CAD/CAM dentistry.

## Introduction

Computer-aided design and computer-aided manufacturing (CAD/CAM) technologies and additive manufacturing are now used for zirconia processing [1, 2]. Zirconia restorations in modern dentistry are often machined from premade blanks using a systematic and reliable production method. Thin restorations for minimally invasive dentistry can be difficult to fabricate using the subtractive approach due to the chipping or cracking of the ceramic material. Furthermore, subtractive manufacturing is frequently accompanied by high cutting tool consumption and large amounts of waste material (zirconia powder) during milling [3]. Also surface treatment and adhesion bonding with primers enhance the durability and longevity of the zirconia [4]. This process is the final step of operative treatment and time consuming, unfortunately there is inadequate resin bond strength [5].

Additive manufacturing (AM) paves the way for developing the manufacturing technology paradigm. High-strength ceramics were created using light-curing additive manufacturing techniques like Stereolithography (SLA) and Digital Light Processing (DLP) [6, 7]. Light-curing of the photosensitive ceramic slurry leads to the formation of layers through the photopolymerization process. Initially, a single layer is created, and with repetition of this step, the three-dimensional body is formed. The green body consists of a three-dimensional crosslinked polymer with ceramic particles trapped inside the polymeric network. Subsequently, the parts undergo cleaning, debinding, and sintering to achieve the final dense ceramic part. This method allows for building complex three-dimensional structures with design freedom and high accuracy to manufacture complex 3D ceramic structures [8–13].

However, the adoption of AM technology is limited by a few technological challenges, one of which is the rough surface texture of AM, which is inferior to that present in manufacturing processes employing conventional technology [14, 15]. Material surface properties influence tribological qualities for dental applications. For the past few years, researchers in the field of dentistry (dental restoration) and orthopaedics (Knee and hip joints) have been focussing on improving the tribological performance, such as generating a textured surface with a lower friction coefficient [16–18]. The effect of generating surface textures on various tribological characteristics, such as adhesive wear, cavitation wear, abrasive wear, elasto hydrodynamic lubrication, and so on, has been extensively studied in the literature. Erosive or attritive processes can cause tooth wear, which can be addressed using minimally invasive techniques to maintain as much healthy dental substance as possible [17]. Occlusal tooth substances can be lost due to extensive tooth wear or erosive compounds. To compensate for the loss of tooth material and to remove the associated symptoms, prosthetic rehabilitation may be required. The employment of additive manufactured dental ceramics as another chair-side machinery has interested researchers during the past few years. From savings on materials and tooling to achieving improved accuracy with the required strength, additive manufacturing could be alternative manufacturing for dental ceramics in the coming years [11, 19–21]. Additive Manufacturing is used in dentistry in various areas, including oral surgery for surgery planning, prosthodontics for prosthesis fabrication, fabrication of fixed and removable appliances and oral prostheses, dental implantology, and orthodontic appliances [22].

However, for applications, a proper understanding of the tribological characteristics of zirconia is a prerequisite. Though several researchers have previously investigated the wear behaviour of CAD/CAM milled zirconia, there are not many reports on the study of tribological properties of additively manufactured structures [23–27]. Hence, a systematic study was undertaken to understand the influence of the fabrication process on the wear behavior and other tribological characteristics of LCM fabricated zirconia specimens. This paper reports the significance of additive manufacturing technology and the evaluation of the tribological characteristics of zirconia specimens.

## Materials and methods

A CeraFab System S65 Medical 3D printer by Lithoz GmbH (Vienna, Austria), was chosen to manufacture 3D-printed ceramic specimens. DLP printing was done from the bottom up using this technology. The machine’s build platform equals 102 × 64 mm² and allows for a maximum print height of 320 mm. A Digital Mirror Device (DMD) projector with a lateral resolution of 1920 × 1080 pixels (X, Y) was integrated to a polymerization LED light with a wavelength of 465 nm. LithaCon 3Y 210 (3 mol percent Yttria-stabilized Zirconia (3Y-TZP)) slurries from Lithoz GmbH (Vienna, Austria) were used.

### Sample Design, Manufacturing, and Thermal Postprocessing

Specimens have been designed with Deskartes 3Data Expert (Deskartes Oy, Helsinki Finland) as cuboids of dimension 10 × 10 × 2 mm³ (X×Y×Z) and exported as STL-file. There were total five samples are prepared for each group.Two print jobs (one with vertical and the other with horizontal orientation of the parts) have been conducted with the parameters presented in Table 1. After finishing the print runs, the parts were removed from the build platform with a razor blade and cleaned within a Cera Cleaning Station Ultra (Lithoz, Vienna, Austria) equipped with LithaSol 30, a proprietary cleaning fluid (Lithoz, Vienna, Austria) and compressed air (according to class 7:4:4 of ISO standard 8753-1:2010). Subsequently, the specimens have been debinded and sintered in a Nabertherm LHTCT 08/16 (Nabertherm, Lilienthal, Germany). To achieve complete density, the sintering has been conducted with a dwell time of 2 h at 1450 °C. No mechanical postprocessing has been conducted – the specimens have been tested “as fired”.

**Table 1:** Printing parameters and materials for manufacturing the zirconia specimens on a CeraFab System S65 Medical

Ceramic Material	LithaCon 3Y 210
Layer height	25 μm
Layer time	36 s
Exposure Intensity Starting Layers	110 mJ/cm²
Exposure Intensity General Layers	110 mJ/cm²
Lateral (X.Y.) shrinking compensation	1.27
Build direction (Z) shrinking compensation	1.30
Z curing depth compensation	Off
Z curing depth compensation layers	0
Contour offset	0 μm
Support structures	None
Vat type	UHC with CeraVat F
Adhesive promoter	LithaFoil
Cleaning Fluid	LithaSol 30

### Sample characterization

For characterization one sample is used from each group. The material characterization was carried out using a Scanning Electron Microscope (SEM) (XL3 FEG, FEI, Eindhoven, Netherlands), that employs a high vacuum source operating at 5 kV voltage and 60 µA current intensity to study the zirconia specimens’ surfaces that were subjected to various surface treatment processes. The X-ray diffraction pattern of the zirconia specimens were obtained using XRD (Rigaku, Japan). The Cu k-α radiation (wavelength = 0.15406 nm) was used to determine the crystalline phase of the surfaces. The scan was performed with a scanning step of 0.02° in the 2θ range of 10–80° to determine the nature of different phases. The 3D-surface roughness (Table 2) of the zirconia specimen was characterized using a non-contact surface roughness tester (Talysurf CCLITE, Magnification of 20X). Three readings were taken at random points on the surfaces of the zirconia specimens in each group. The Vickers’s Micro hardness test was carried out under a 1 kg load for 15 s using HMV-G series, Shimadzu, Kyoto, Japan with five indentations per sample.

**Table 2:** Surface Roughness parameters abbreviation

S.no	Roughness parameters	Symbol
1.	Average roughness	Sa (µm)
2.	Root mean square roughness	Sq (µm)
3.	Skewness	Ssk
4.	Kurtosis	Sku
5.	Maximum peak height	Sp (µm)
6.	Maximum valley depth	Sv (µm)
7.	Maximum height of surface	Sz (µm)
8.	Texture aspect ratio	Str
9.	Auto correction Length	Sal (µm)
10.	Texture direction	Std (°)
11.	Peak material volume	Vmp (µm³/µm²)
12.	Core material volume	Vmc (µm³/µm²)
13.	Core void volume	Vvc (µm³/µm²)
14.	Dale void volume	Vvv (µm³/µm²)
15.	Reduced peak Height	Spk (µm)
16.	Core roughness depth	Sk (µm)
17.	Peak material portion	Smr1 (%)
18.	Reduced valley depth	Svk (µm)
19.	Valley Material portion	Smr2 (%)

### Parameters in Tribology Test

The investigation of the wear behaviour was conducted on the Y-TZP specimens with a ZrO_2_ ball acting as the antagonist mounted on a reciprocating ball-on-plate tribometer (Fretting machine, Ducom, India). The wear behaviour of four samples from each group was studied. The antagonist Zirconia ball had a diameter of 6 mm, while the block dimension of the zirconia plate was 10 × 10 × 2 mm³. The wear test parameters were evaluated under a constant applied load of 10 N at a temperature of 37 ± 1°, with 0.5 mm stroke length, for a duration of 1 h at a frequency of 5 Hz. Distilled water (1000 ml) was used to prepare the artificial saliva medium [25]. The frictional force recorded during the test was subsequently used to determine the friction coefficient.

## Results

### SEM and 3-D surface topography

**Fig. 1:**
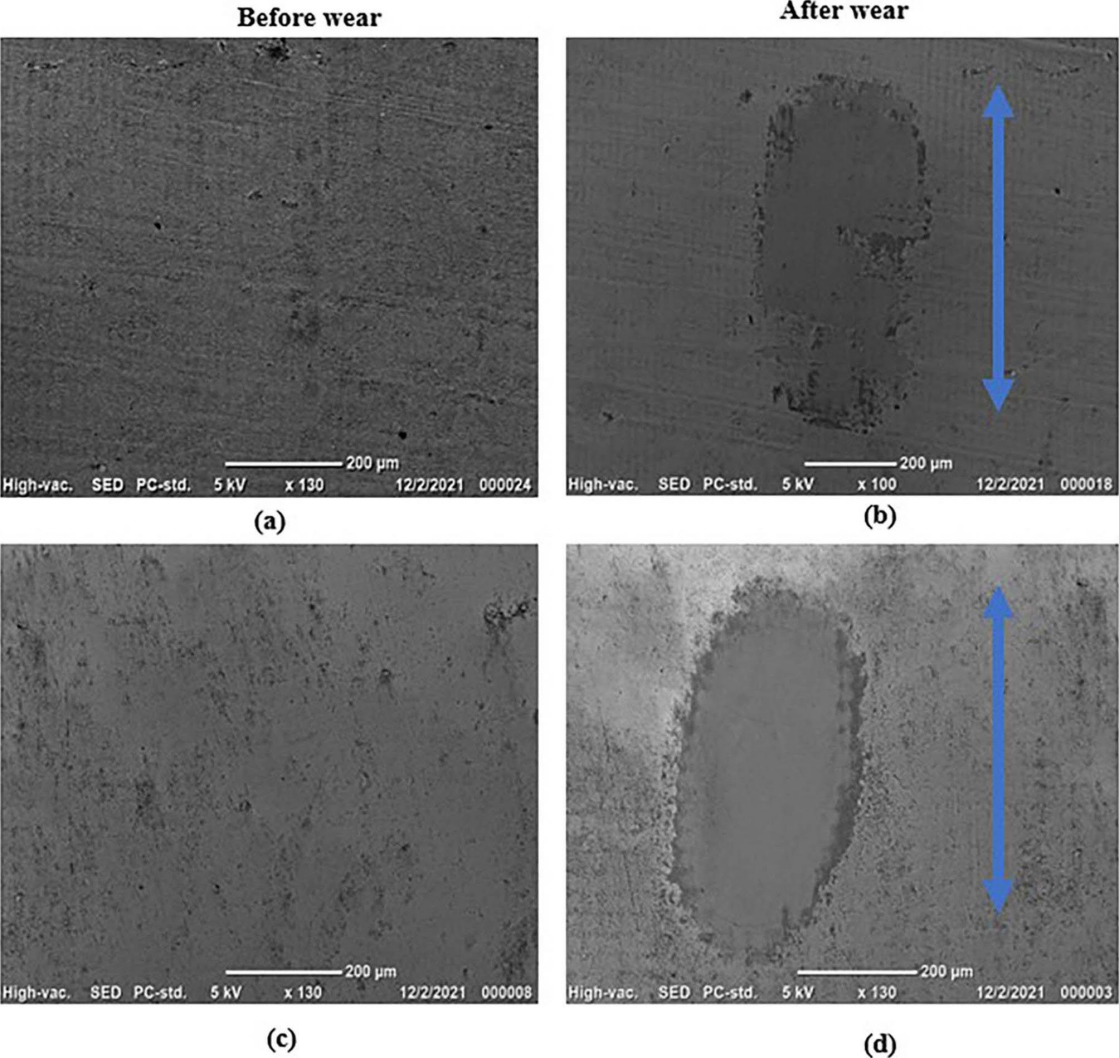
SEM images of the (a & b) vertically printed and (c & d) horizontally printed zirconia specimens at a magnification of 130X before and after wear

**Fig. 2:**
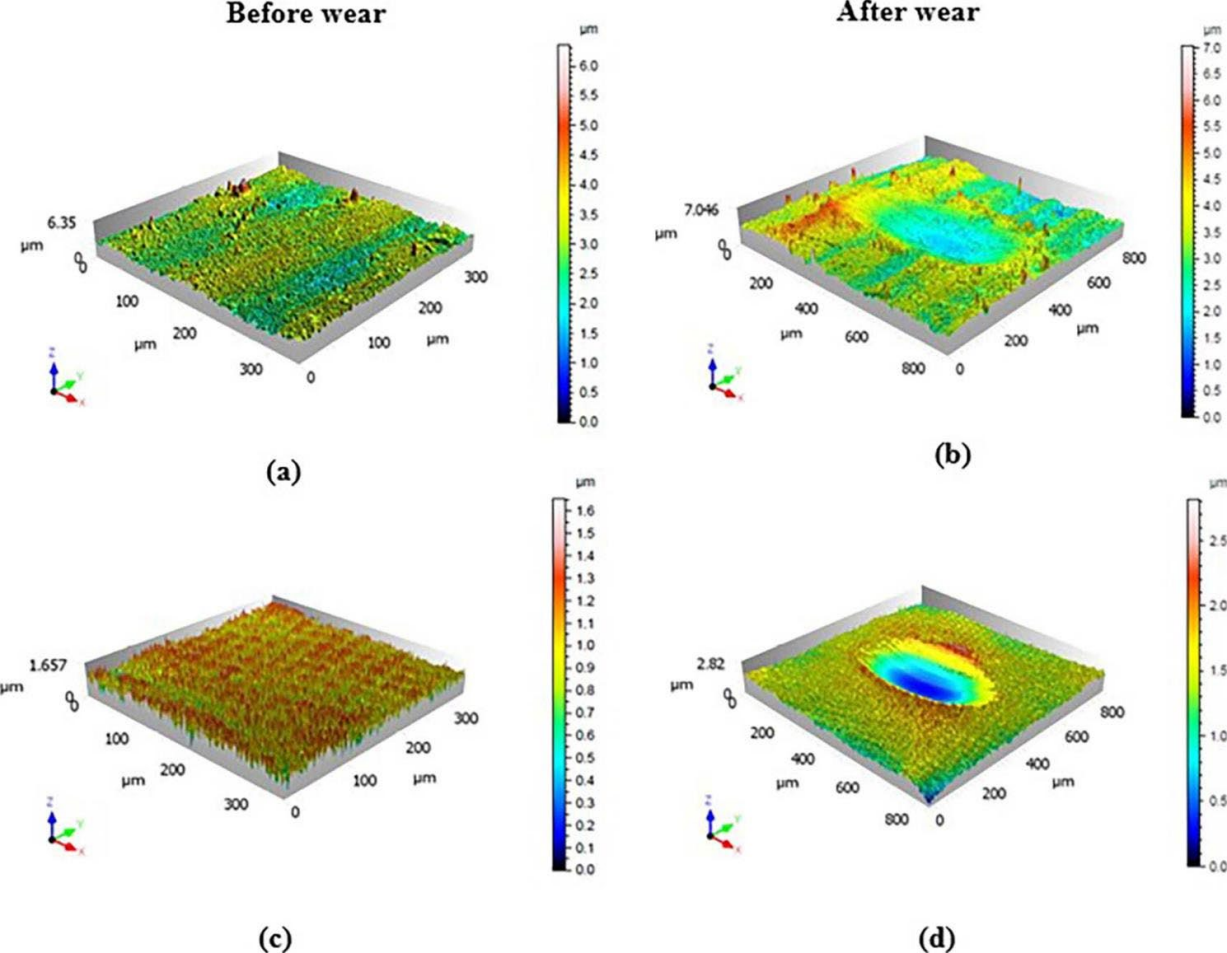
3D topographic profiles of (a & b) vertically printed and (c & d) horizontally printed zirconia specimens, before and after wear

SEM images with the 3D topography of the vertically printed (V.P) and horizontally printed (H.P) micropores on the zirconia specimens are shown in Fig. 1 (a) and (c) and Fig. 2 (a) and (c), respectively. The micropores are usually less than 2 nm in size. The surface was observed to be uneven, but the groves were absent. After subjection to wear, the wear track was observed on the specimen. Arrows represent the direction of motion of the wear in Fig. 1 (b) & (d). The sample with vertically printed micropores has less wear scar compared to the horizontally printed one. This observation is even more evident from the 3D–topography results in Fig. 2 (b) and (d). This qualitative representation was further quantified by determining the 3D–surface roughness values. The notable observation is the decline of 3D–surface roughness values after wear. The reduced valley depth (Svk) and the dale void volume (Vvv) were low for the vertically printed zirconia specimen, indicating lesser voids and low fluid retention.

### Hardness value

**Fig. 3:**
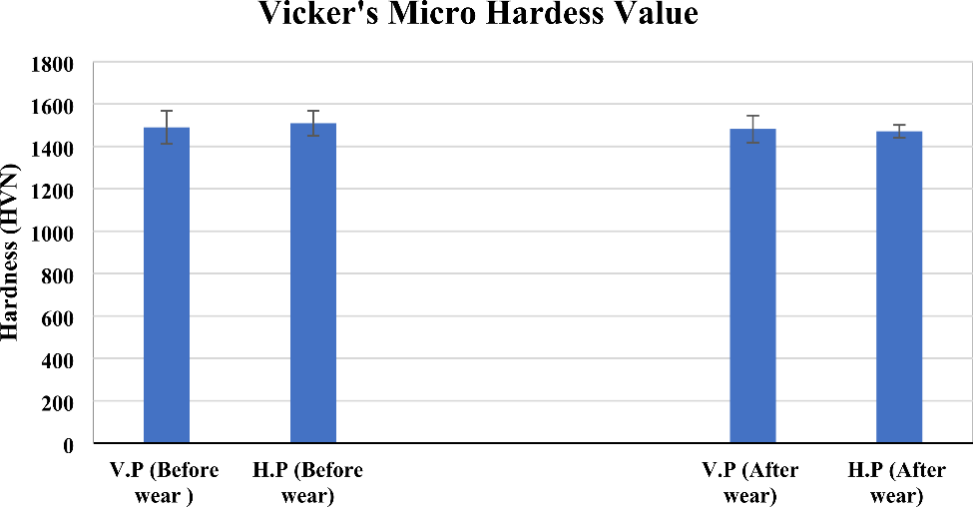
Micro Vickers’s hardness test results of the horizontally and vertically printed zirconia specimens, before and after wear

The results of the Vickers microhardness test in Fig. 3 show a decline in the hardness values after wear for both the horizontally and vertically printed zirconia specimens. The percentage decline in the hardness value was about 0.5% for the vertically printed and 2.5% for the horizontally printed samples.

### Tribology

**Fig. 4:**
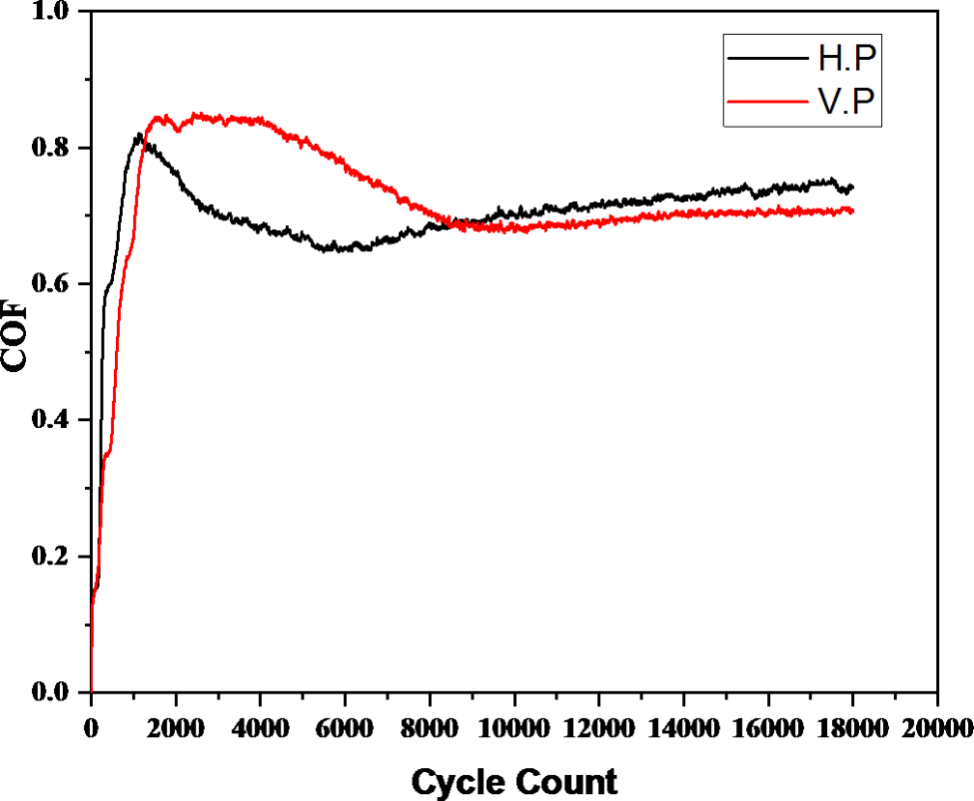
Coefficient of friction (COF) of the vertically and horizontally printed zirconia specimens

**Fig. 5:**
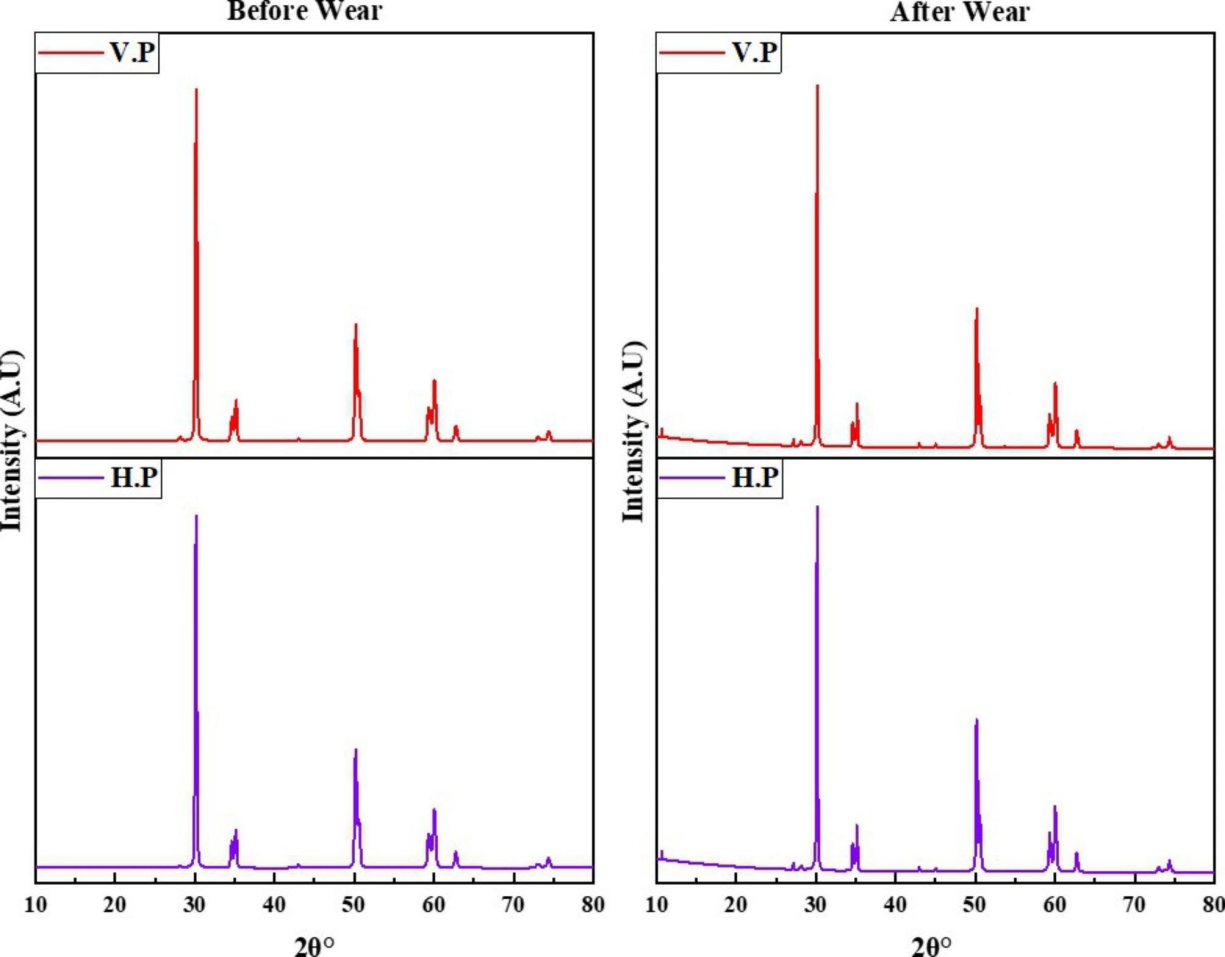
XRD patterns of vertically and horizontally printed zirconia specimens, before and after wear

During the initial cycles, the coefficient of friction (COF) for both the vertically and horizontally printed specimens was observed to increase, reach a maximum, and then decrease to attain a steady-state regime (Fig. 4). The steady-state value of the COF was around 0.7 for both the V.P and H.P specimens. However, the SEM images and 3D profiles of the specimens after wear (Fig. 1 (b) & (d), Fig. 2 (b) & (d)) reveal that the material is worn out and the direction of the motion is along the wear track. It was observed that the wear damage was more uniform in the case of horizontally printed specimens when compared to that of vertically printed samples. The wear scars were more elliptical in the horizontally printed material (Fig. 1(b) & Fig. 2 (b)). Thus, the COF was studied to measure the wear of the material. The XRD patterns of the specimens after wear reveal a monoclinic phase with the characteristic peak observed at a 2θ value of 28.1°, as seen in Fig. 5. This indicates that wear affects the crystal structure of the zirconia specimens.

## Discussion

Ceramic additive manufacturing involves the bonding process, both within and in between layers, is caused by chemical reaction of the organic binder (i.e. radical photopolymerization) The bonding depends on light intensity, time period allowed during irradiation as well as the reactivity of the binder formulation of the ceramic suspension. Other factors that affect bonding are the homogeneity of the ceramic particles within and between the layers and the solid loading, indicating green density. These parameters have a role for wear characteristics too.

The SEM images in Fig. 1 (a) and (c) reveal the microporosity at the surface layer. However, after wear, the abraded layer or wear crater has no microporosity, as seen in Fig. 1 (b) and (d). Thus, the process of wear is thought to have led to the development of debris of ceramics. The softer material gets abraded easily. Hence, a comparison of Fig. 1(b) & (d) shows that the horizontally printed material has deeper wear scars than the vertically printed specimen. The results of the microhardness tests on the zirconia specimens shown in Fig. 3 clearly show that the horizontally printed material has a lower hardness value (1471 ± 29.60 HVN) after wear. It was noted that in Fig. 5, the monoclinic transformation has less impact on the material stability. There was no fracture or rupture of the material.

Surface roughness gives information on wear, lubrication, and component lifespan prediction, among other things. Several standards for evaluating the two-dimensional and three-dimensional surface roughness have been created and are widely used [28–30]. The data on wear can be studied deeper with the help of 3D-parameters listed in Table 3.

**Table 3:** 3D–Surface roughness parameters of the horizontally (H.P) and vertically (V.P) printed zirconia specimens before (b.w) and after wear (a.w)

Height	H.P (b.w)	Std. dev for H.P (b.w)	H.P	Std.dev for H.P (a.w)	V.P	Std. dev for V.P	V.P	Std. dev for V.P
			(a.w)		(b.w)	(b.w)	(a.w)	(a.w)
Sq	0.988	0.013	0.824	0.15	0.693	0.034	0.509	0.141
Ssk	-1.44	0.176	-1.426	0.091	-0.119	0.023	-0.584	0.056
Sku	5.07	0.498	8.481	2.842	6.115	0.184	7.168	1.852
Sp	4.714	0.696	2.414	0.45	5.484	0.999	2.565	0.054
Sv	4.801	0.319	5.175	0.182	4.003	0.167	3.919	0.435
Sz	8.515	0.407	7.59	1.472	9.488	1.175	6.485	1.463
Sa	0.747	0.012	0.567	0.039	0.525	0.011	0.379	0.02
Functional								
Vmp	0.039	0.003	0.019	0.002	0.034	0.002	0.026	0.003
Vmc	0.823	0.018	0.535	0.078	0.57	0.016	0.411	0.075
Vvc	0.728	0.034	0.669	0.029	0.733	0.031	0.534	0.066
Vvv	0.2	0.012	0.156	0.013	0.09	0.012	0.066	0.018
Functional								
Sk	1.426	0.096	1.37	0.197	1.591	0.042	1.146	0.176
Spk	0.759	0.086	0.383	0.055	0.678	0.051	0.532	0.034
Svk	2.046	0.108	1.642	0.143	0.9	0.194	0.665	0.143
Smrk1	9.555	0.153	5.585	1.21	10.587	0.672	9.371	1.062
Smrk2	75.187	0.813	84.144	3.527	89.2	0.568	88.974	1.732

In the 3D–printing method, the surface texture forms naturally during the process of transfer, wherein exposure to light radiation causes the liquid used in the stereolithography process to harden in a layer by layer manner until the desired shape is achieved. The visible light radiation induces a polymerization process in which the polymerizable components present in the suspension combine together and solidify, thereby allowing them to be cast into a 3D object. The surface texture in this study is called micro-texture, since the peak (Sp, Spk) and valley (Sv, Svk) dimensions and the layer heights are much smaller than that observed in subtractive manufacturing. In the present study, when the surface layer was abraded, the surface roughness reduced, with the Sa values for H.P and V.P being 0.567 ± 0.139 μm and 0.379 ± 0.080 μm, respectively. The surfaces became smooth after wear, as seen in Fig. 2 (b) and (d) and Table 3 as compared with before wear as shown in Fig. 2 (a) and (c). The hardness of the LCM fabricated zirconia specimens in the present study was around 14 HVN and was almost similar to that obtained by the conventional manufacturing process.

Anna Paradowska-Stolarz et al. [31] studied the texture and mechanical properties of 3-D printed resin and showed less texture changes. The development of surface texture such as ceramic grooves, micro-dimples, microchannels, micro-grids, and micro-pores on the material’s surface using 3D-printing technology would be of significant interest in studying the nature of the material surface. The micro void volume parameters Vvv and Svk from Table 3 indicate that the wear scar or the valley depth on the specimens is shallow compared to that observed in subtractive manufacturing [23]. In addition, no deeper machining traces or scars were observed in 3D-printed zirconia. However, the direction of printing could be observed. Using additive manufacturing to create surface roughness has a clear advantage over the traditional surface preparation method of removing material by scraping the surface. Unlike in other technologies, surface preparation in AM occurs naturally during production. Surface preparation by secondary operations is not required after manufacturing the material. All kinds of geometric surface textures may be made in detail with the required precision permitted by AM during the manufacturing process, which is essential for surface integrity and application time and costs.

The compression and tensile study on the effect 3D printed resin and concluded the resistant tensility test [32]. The author studied the effect of thermocycling on the bond strength of adhesive cement and resulted in decrease of bond strength [33]. The surface roughness greatly influences bonding of the material. According to [34], feldspathic ceramic crowns that are hard milled have greater surface roughness in comparison to soft-milled crowns made of zirconia. Compared to zirconia crowns, degradation has a more significant impact on the roughness of the feldspathic ceramic crown surfaces. In the present study, the surface roughness obtained was 0.7 μm, indicating that clinically acceptable values for minimal surface roughness of dental restorations could be achieved using zirconia fabricated by Lithoz’s LCM technology.

Material surface qualities have a significant impact on their tribological properties. Creating a textured surface while improving tribological performance that include achieving reduced coefficient of friction and increased resistance to wear, has been gaining significant attention in recent decades. In the present study, the measurement of the friction coefficient, as in Fig. 4, showed that the friction torque changed significantly at the beginning, which is linked to the start of the experiment. Further, the process of running in the tribopair (Zirconia) illustrated in Fig. 4 is caused due to the plastic flow of the material.

From Fig. 4, it is noticed that the COF of both the vertically and the horizontally printed specimens are approximately 0.7. When two surfaces with asperities meet, they start touching at the highest points. Since the usual force at first contact is provided by only a small region, the local pressure is exceptionally high (load). Therefore, the tallest asperities deform depending on the magnitude of the normal force until enough bearing area has grown to carry the load.

During tribology measurements, high points on the surfaces come into contact during wear or friction. The shape, form, and deformation qualities of the asperities play a role in tribology contact modelling. The Spk (reduced peak height) of H.P and V.P specimens predominately decreased after wear, as seen in Table 3. The percentage of peak material Smr1 reduces by 10% after wear. Depending on the phenomena under discussion, asperities on contact surfaces can be viewed at several dimensional scales (Sp, Spk, Vmp). Breakage, deformation, and/or loss of roughness are all linked to wear, polishing, and roughening or smoothing of surfaces.

Limitation of the study is that only zirconia by LCM technology and thickness of layer height was 25 μm is studied. The influence of layer orientation of different thicknesses on the tribological properties of 3D-printed parts made of a different material may be investigated in the future.

## Conclusion

For organizations aiming to enhance manufacturing efficiency, AM opens up new possibilities and lends itself to several alternatives. AM simplifies the traditional processes greatly and has the potential to become the standard in the coming decade. The investigation of the tribological characteristics of additively manufactured zirconia shows that the volumetric substance loss was negligible in the specimens subjected to occlusal wear by zirconia antagonists. Both the H.P and V.P specimens exhibited a friction coefficient of 0.7. Thus, both materials exhibit better resistance to wear. The results of the present study show similarity to those from the CAD/CAM milling process.

Further evaluation revealed that the 3D surface roughness before and after wear ranges between 0.168 and 0.747 μm, much lower compared to subtractive manufacturing of zirconia. The direction of printing influences the material’s properties and hence must be considered when planning a printing strategy. The 3D roughness parameters like reduced valley depth (Svk) and the dale void volume (Vvv) were low for the vertically printed zirconia specimen, indicating lesser voids and low fluid retention that makes the printing process as an alternate chair side manufacturing for dentistry.

## Data Availability

Corresponding author will share required data as and when requested by the reviewers.
